# Mitochondrial DNA variant m.15218A > G in Finnish epilepsy patients who have maternal relatives with epilepsy, sensorineural hearing impairment or diabetes mellitus

**DOI:** 10.1186/1471-2350-14-73

**Published:** 2013-07-19

**Authors:** Heidi K Soini, Jukka S Moilanen, Tiina Vilmi-Kerälä, Saara Finnilä, Kari Majamaa

**Affiliations:** 1 Department of Neurology, Oulu University Hospital, P.O. Box 20, Oulu FI-90029, OYS, Finland; 2 Department of Clinical Medicine, Neurology, University of Oulu, P.O. Box 5000, FI-90014, Oulu, Finland; 3 Clinical Research Center, Oulu University Hospital, P.O. Box 5000, FI-90014, Oulu, Finland; 4 Department of Clinical Genetics, Oulu University Hospital and University of Oulu P.O. Box 23, FI-90029, OYS, Oulu, Finland

**Keywords:** Epilepsy, mtDNA, Mitochondrial DNA, Mitochondrial haplogroups, Sequence variation, Nonsynonymous mutation, Maternal inheritance, Haplogroup U5a1

## Abstract

**Background:**

Mitochondrial diseases caused by mutations in mitochondrial DNA (mtDNA) affect tissues with high energy demand. Epilepsy is one of the manifestations of mitochondrial dysfunction when the brain is affected. We have studied here 79 Finnish patients with epilepsy and who have maternal first- or second-degree relatives with epilepsy, sensorineural hearing impairment or diabetes mellitus.

**Methods:**

The entire mtDNA was studied by using conformation sensitive gel electrophoresis and PCR fragments that differed in mobility were directly sequenced.

**Results:**

We found a common nonsynonymous variant m.15218A > G (p.T158A, *MTCYB*) that occurs in haplogroup U5a1 to be more frequent in patients with epilepsy. The m.15218A > G variant was present in five patients with epilepsy and in four out of 403 population controls (*p* = 0.0077). This variant was present in two branches in the phylogenetic network constructed on the basis of mtDNA variation among the patients. Three algorithms predicted that m.15218A > G is damaging in effect.

**Conclusions:**

We suggest that the m.15218A > G variant is mildly deleterious and that mtDNA involvement should be considered in patients with epilepsy and who have a maternal history of epilepsy, sensorineural hearing impairment or diabetes mellitus.

## Background

Epilepsies are a heterogeneous group of complex seizure disorders, which can be classified according to underlying pathology or clinical manifestation. Main classes of epilepsy in adults are localization related (focal, partial), generalized, undetermined and epilepsies associated with special syndromes. Causes for epilepsy include structural damage of brain tissue such as trauma, stroke and tumours, central nervous system infections and genetic causes [1,2]. Some epilepsy cases remain etiologically undetermined.

Epilepsy is a frequent manifestation of mitochondrial disorders, as the brain is often severely affected in these diseases [3]. Seizures are commonly seen in Alpers-Huttenlocher syndrome, Leigh syndrome, MELAS syndrome (mitochondrial encephalomyopathy, lactic acidosis and stroke-like episodes), MEMSA syndrome (myoclonic epilepsy, myopathy, sensory ataxia) and in ataxia neuropathy spectrum [4,5]. The most common mitochondrial DNA (mtDNA) mutation causing epilepsy is m.8344A > G in the *MTTK* gene. It causes MERRF syndrome (myoclonus epilepsy associated with ragged red fibers) [6]. Mutations in the *MTTF* gene have also been reported to cause severe epilepsy [7]. Pathogenic mtDNA mutations are often heteroplasmic so that both the mutant variant and the wild type variant are present in the mitochondria. The proportion of the mutant variant must exceed a certain threshold before symptoms of mitochondrial disease become manifest [8]. Other mitochondrial diseases which can present with seizure symptoms are neuropathy, ataxia, retinitis pigmentosa syndrome and Kearns-Sayre syndrome [4]. Furthermore, mitochondrial oxidative stress may play a role in epileptogenesis by virtue of affecting neuronal excitability [9].

It has been suggested that mildly deleterious mtDNA variants are involved in the pathogenesis of many degenerative diseases [10-12]. Such variants are present in the population without being subjected to selection [13], but are maintained more by genetic drift [14]. The increased risk for diseases with mitochondrial contribution in pathogenesis is most likely determined by a balance between beneficial and damaging mtDNA mutations within haplogroups. In the case of some haplogroups, the balance leans more towards the deleterious side. Also, deleterious mtDNA mutations have been suggested to be population specific so that a given variant may be associated with a disease in one population, but not in the other [14].

Deleterious mtDNA mutations are the cause of the MELAS syndrome [15] and the MERRF syndrome [16]. In addition to the full-blown encephalomyopathic MELAS syndrome, patients with the m.3243A > G mutation often present with diabetes mellitus or sensorineural hearing impairment (SNHI) [17]. These two phenotypes as well as epilepsy are common in the population and, therefore, patients with these diseases are good subjects in the search of mildly deleterious mtDNA variants. In order to assess the role of such variants and mtDNA haplogroups we determined the entire mtDNA sequence of 79 Finnish patients with epilepsy and with a maternal history of epilepsy, diabetes mellitus or SNHI. The pathogenic potential of all nonsynonymous mutations was also analysed.

## Methods

### Subjects and samples

Most adult patients with epilepsy in the province of Northern Ostrobothnia make regular visits to the outpatient Neurology Department of the Oulu University Hospital. During a one year period, a physician involved in the study reviewed the charts of the visiting patients and once the diagnosis of epilepsy was confirmed, the patient was requested to fill out a family history questionnaire. We did not make distinctions between the etiologies or types of epilepsy. We identified 223 patients with epilepsy that had any combination of epilepsy, sensorineural hearing impairment or diabetes mellitus in their first- or second-degree maternal relatives. Blood samples were obtained from 165 patients and then 79 patients were selected if the number of maternal relatives with epilepsy, diabetes or sensorineural hearing impairment was ≥ 2 (38 patients) or if a family history score was ≥ 0.1 in families with one maternal relative with epilepsy or sensorineural hearing impairment (41 patients). The family history score was calculated using the formula N_affected_/N_total_[18]. Patients were tested not to harbour m.3243A > G or m.8344A > G mutation using restriction fragment analysis prior to selecting samples for this study.

Samples from healthy blood donors (N = 403) obtained at local Finnish Red Cross offices served as population controls [19]. It was required that the donors did not report neurological ailments, diabetes and sensorineural hearing impairment of their own or of their mothers. It was also required that the donor and the mother had been born in the same province. All the patients signed an informed consent to participate in the study. The Ethics Committee of the Medical Faculty, University of Oulu, and that of Finnish Red Cross have approved the study protocol.

### Conformation Sensitive Gel Electrophoresis (CSGE)

Total DNA was extracted from blood using the QIAmp Blood Kit (Qiagen, Valencia, CA, U.S.A.). Mitochondrial DNA haplogroups were determined by restriction fragment analysis [20]. CSGE was performed as described earlier [21,22]. MtDNA coding region (nucleotides m.577-16090) was amplified in 63 partially overlapping fragments covering the entire mtDNA coding region. The primers were ~22 bp in length and they were designed as previously reported [21]. The mean PCR fragment size was 350 bp and each amplified fragment overlapped by about 80 nucleotides with its neighbouring fragments [see Additional file 1]. PCR fragments were amplified in a total volume of 30 μl in 30 cycles: denaturation at 94˚C for 1 min, annealing at a primer-specific temperature and extension at 72˚C for 1 min and a final extension for 10 min. Touchdown-PCR protocol was used in parallel and yielded similar results. 3-10 μl of the PCR product was used for heteroduplex formation. Each amplified fragment was mixed with a reference sample and denatured at 95˚C for 5 min and annealed at 68˚C for 30 min for heteroduplex formation. Samples were electrophoresed on 15% polyacrylamide gel overnight at a constant voltage of 400 V at room temperature. The gel was stained in 150 μg/l of ethidium bromide for 5 min and destained in water after which it was transferred to UV transluminator and photographed (Grab-IT Annotating Grabber 2.04.7; UVP Inc. Upland, CA, U.S.A.). Previous data have suggested that the limit of detection of heteroplasmy in various heteroduplex screening assays is < 10% [23].

### Sequencing

PCR fragments that differed in mobility in CSGE were purified with *exonuclease I* and shrimp alkaline phosphatase [24] and sequenced (ABI PRISM™ 377 and ABI PRISM™ 3100 Sequencers using DYEnamic ET Terminator Cycle Sequencing Kit; Amersham Pharmacia Biotech Inc. Piscataway, NJ, U.S.A.). Same primers were used for sequencing and amplification of the 63 PCR fragments. CSGE has previously been shown to be a sensitive (98.8%) and specific (100%) method for detecting mtDNA variants [25]. The D-loop (nucleotides m.15975 – 725) was amplified in a unique PCR fragment and directly sequenced.

### Analysis of substitutions

Sequences were compared to the revised Cambridge reference sequence NC_012920 [26] and to mtDNA sequences available in the GiiB-JST mtSNP database [http://mtsnp.tmig.or.jp/mtsnp/index_e.shtml] [27], mtDB Human Mitochondrial Genome database [http://www.mtdb.igp.uu.se] [28], HmtDB database [http://www.hmtdb.uniba.it] [29] and Mitomap [http://www.mitomap.org] [30] accessed in June 2012. Nonsynonymous polymorphisms were considered rare if a total of three or less sequences had been reported in the above mentioned databases. Sequences were also compared to complete mtDNA sequences of 192 population controls obtained from the same area as the patients [19,31].

Novel substitutions were confirmed by restriction fragment analysis and/or sequencing in both directions at least twice from separate PCR products. Variant was regarded as a previously reported pathogenic mutation if it was listed as such in the Mitomap. Base conservation was determined using the GiiB-JST mtSAP evaluation http://mtsnp.tmig.or.jp/cgi-bin/mtsnp/specAlign/ctrlSpecAlignE.cgi for nonsynonymous variants [27] and Mamit-tRNA for tRNA variants http://mamit-trna.u-strasbg.fr/[32]. Phylogenetic networks based on mtDNA sequence variation in the coding region and the D-loop were constructed using the median algorithm [33].

### Analysis of pathogenic potential of nonsynonymous mtDNA variants

The pathogenic potential of nonsynonymous mtDNA variants detected in epilepsy patients was analyzed with PolyPhen-2, version 2.2.2 [http://genetics.bwh.harvard.edu/pph2/] [34], SIFT BLink [http://sift.jcvi.org/www/SIFT_BLink_submit.html] [35] and PMut [http://mmb.pcb.ub.es] [36]. PolyPhen-2 uses a Bayesian algorithm to calculate the pathogenic potential of a nonsynonymous mutation using both evolutionary conservation data and the structural similarities/differences of amino acids. The mutations are classified as benign (< 50% chance of pathogenic effect), possibly deleterious (> 50% chance of pathogenic effect) and probably deleterious (> 90% chance of pathogenic effect). SIFT BLink uses evolutionary conservation data to assess whether a nonsynonymous variant is pathogenic. It predicts a variant to be either functionally important (0 being the most pathogenic) or unimportant (1 being the most benign). PMut is based on evolutionary information derived from multiple sequence alignments. Variants are classified as pathogenic or benign and a reliability score is given (0, lowest reliable; 9, very reliable).

## Results

### Nonsynonymous variants predicted to be damaging

**Figure 1:**
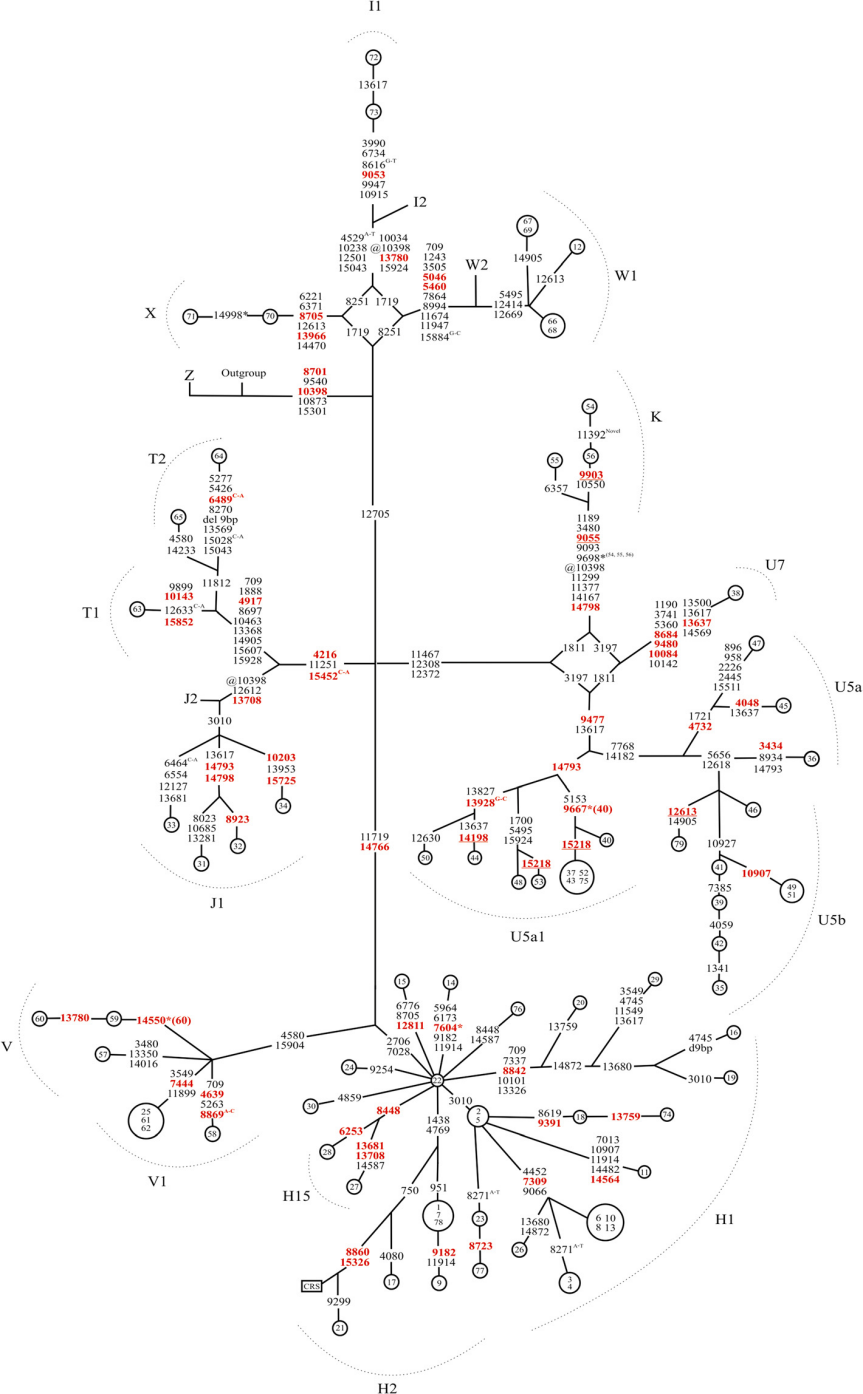
**Phylogenetic network based on mtDNA coding region sequences in 79 patients with epilepsy and with a maternal history of epilepsy, SNHI or diabetes mellitus.** Nodes represent patients. Inside the nodes, patients are identified by numbers 1-79. Outgroup is an African sequence [GenBank: AF346980]; CRS is the revised Cambridge Reference Sequence [GenBank: NC_012920]. Unless marked otherwise, mtDNA variants are transitions. Superscripts indicate transversions and novel mutations. @ = back mutation, * = heteroplasmic mutation, followed by patient number in parenthesis if required. D9bp = deletion spanning the positions m.8281 and m.8289. Nonsynonymous variants are in red font, underlined red variants were concordantly predicted to be pathogenic by PolyPhen-2, PMut and SIFT BLink analyses.

**Figure 2:**
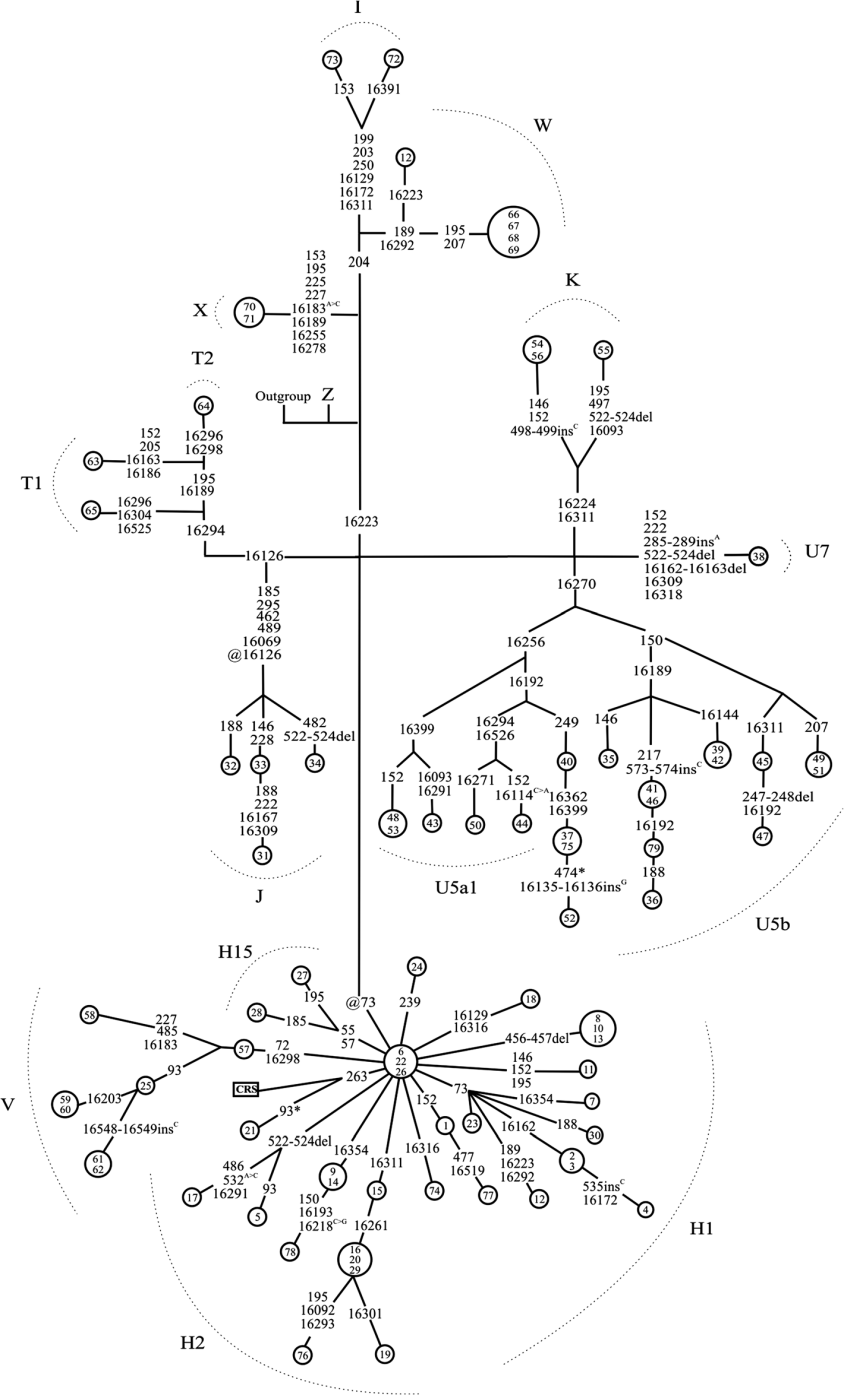
**Phylogenetic network based on mtDNA D-loop sequences in 79 patients with epilepsy.** Nodes represent patients. Inside the nodes, patients are identified by numbers 1-79. Outgroup is an African sequence [GenBank: AF346980]; CRS is the revised Cambridge Reference Sequence [GenBank: NC_012920]. Unless marked otherwise, mtDNA variants are transitions. Superscripts indicate transversions and novel mutations. @ = back mutation, * = heteroplasmic mutation.

CSGE and sequencing were used to determine complete mtDNA sequences of 79 patients with epilepsy. MtDNA sequences were then used to construct a phylogenetic network separately for the coding region (Figure 1) and the D-loop (Figure 2). Exact test of population differentiation [38] revealed no differences in the frequencies of mtDNA haplogroups between patients and controls (Table 1).

**Table 1:** Frequencies of mtDNA haplogroups in 79 Finnish patients with epilepsy and in 403 controls

	**Patients**		**Controls**^**1**^	
**Haplogroup**	**(N)**	**(%)**	**(N)**	**(%)**
H	32	25.3	162	40.1
U	21	16.6	112	27.8
V	7	5.5	22	5.4
W	5	3.9	37	9.2
J	4	3.2	18	4.4
K	3	2.4	12	2.9
T	3	2.4	10	2.5
I	2	1.6	14	3.5
X	2	1.6	4	0.9
M	0	0	10	2.5
Other	0	0	1	0.2

^1^Meinilä et al. 2001 [19].

**Figure 3:**
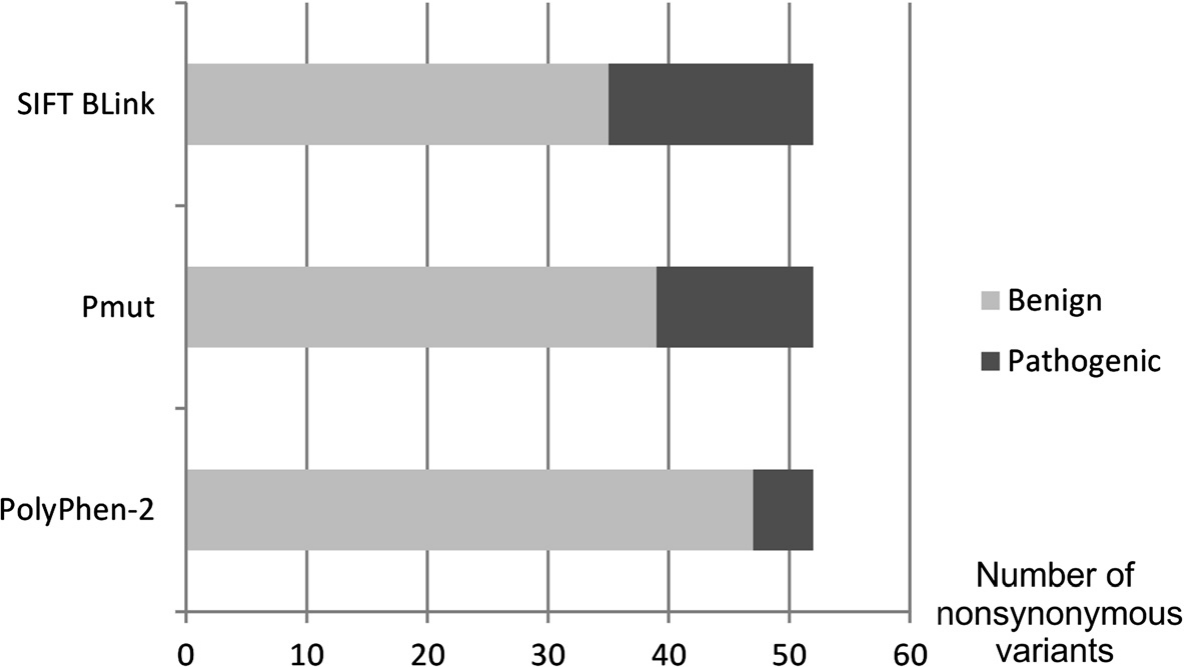
**Number of nonsynonymous variants predicted to be pathogenic or benign in 79 patients with epilepsy. **Pathogenicity predictions were carried out on 52 nonsynonymous mtDNA mutations in 79 patients with epilepsy by using PolyPhen-2, SIFT BLink and PMut analyses. Gray bar, number of variants predicted to be benign; black bar, number of variants predicted to be pathogenic.

**Figure 4:**
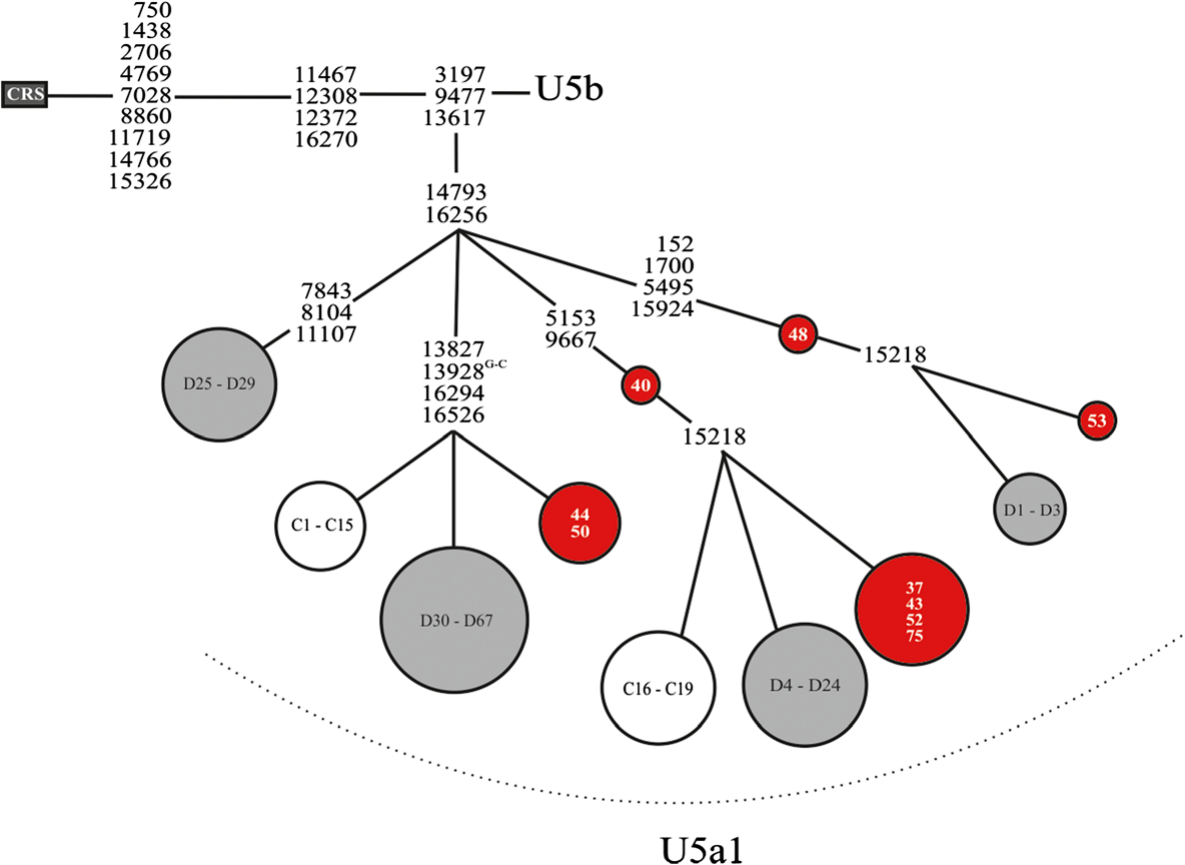
**Schematic presentation of haplotypes in mtDNA haplogroup U5a1. **Red nodes, patients with epilepsy (N = 9); white nodes, Finnish population controls (N = 19); grey nodes, sequences found in HmtDB database (N = 66). Inside the nodes, patients identified by numbers; population controls by numbers C1 - C21 [19], database controls by numbers D1 – D66. Only discriminant variants for the four haplotypes are shown. CRS, the revised Cambridge Reference Sequence [GenBank: NC_012920]. Unless marked otherwise, variants are transitions. GenBank accession for database controls; D1 - D3: AY339528, AY339529, EF657601; D4 – D24: GU296581, GU296583, GU296594, GU296558, HM852852, GU459066, HM852873, DQ489510, GQ160809, GU296652, GU296636, GU296601, GU296595, EU597527, EU698951, GQ368895, GU206811, EU523128, DQ904330, DQ826448, EF657412; D25 - D29: AY339523, DQ112838, EF397754, HM490393, JN604831; D30 - D66: AY339524, AY339525, AY339526, AY339527, EF657616, EF363686, EF177408, EU215455, GU391321, GQ214520, HM246245, HM229344, GU122993, HM144108, HM142902, GU296650, GU296635, GU296634, GU296626, GU296615, GU296613, GU296605, GU296602, GU296597, GU296596, GU296580, GU296574, GU296548, GU797137, EU007851, EU124886, AM260578, AM260577, AM260576, AM260573, AM260572, HM765474.

We found 52 different nonsynonymous mtDNA variants in the 79 patients. Evaluation of the pathogenic potential of all the nonsynonymous variants was then carried out by using PolyPhen-2, SIFT BLink and PMut. PolyPhen-2 predicted the lowest and SIFT BLink the highest number of pathogenic mutations (Figure 3). All three algorithms concordantly predicted five nonsynonymous variants to be damaging (Table 2). One of these variants was m.15218A > G (p.T158A, *MTCYB*), which was present in two mtDNA haplotypes in five patients (Figure 4). Four out of 403 population controls belonged to the major haplotype while none belonged to the minor haplotype (*p* = 0.0077 for difference between cases and controls, Fisher’s exact two-tailed test). The presence of m.15218A > G in the five patients and four controls and the absence of the variant in samples 40 and 48 was confirmed by sequencing the fragment amplified in two separate reactions in both L- and H-directions. Patients 37 and 75 with m.15218A > G had identical mtDNA sequences, but family information did not suggest immediate relatedness. The clinical features of the five patients were variable (Table 3). Another variant predicted to be damaging was m.9903T > C (p.F233L, *MTCO3*) that occurred in two patients but not in the controls (*p* = 0.08, Fisher’s exact two-tailed test). The remaining three variants did not differ in frequency between patients and controls.

**Table 2:** Nonsynonymous mtDNA variants predicted to be deleterious in 79 patients with epilepsy

**Variant**	**Gene**	**Amino acid change**	**PolyPhen (%)**^**1**^	**SIFT Blink**^**2**^	**PMut prediction**^**3**^	**PMut reliability score**^**4**^	**Database hits**^**5**^	**Source**
m.9055G > A	*MTATP6*	p.A177T	84.5	0.01	Pathogenic	7	446	Various
m.9903T > C	*MTCO3*	p.F233L	75.6	0	Pathogenic	7	4	Africa, Finland, Italy^6^
m.12613G > A	*MTND5*	p.A93T	97.2	0	Pathogenic	2	5	Finland, Russia/Tatar
m.14198G > A	*MTND6*	p.T159M	99.9	0	Pathogenic	0	5	Finland, Japan, Israel, Spain^7^
m.15218A > G	*MTCYB*	p.T158A	89.3	0.04	Pathogenic	2	164	Various

^1^http://genetics.bwh.harvard.edu/pph2/ PolyPhen-2 pathogenicity prediction levels: Possibly damaging > 50%, probably damaging > 90%.

^2^http://sift.jcvi.org/www/SIFT_BLink_submit.html SIFT BLink pathogenicity prediction levels: Damaging amino acid substitutions ≤ 0.05.

^3^http://mmb.pcb.ub.es.

^4^ PMut pathogenicity prediction reliability score, 0 (lowest reliable) to 9 (most reliable).

^5^ Number of sequences among 2704 sequences in mtDB database http://www.mtdb.igp.uu.se/ and among 8813 sequences in HmtDB database http://www.hmtdb.uniba.it accessed in February 2013, each sequence hit was counted only once if present in both databases.

^6^ Patient with breast cancer [38].

^7^ Patient with glioma [39].

**Table 3:** Clinical features of patients with epilepsy and with m.15218A > G

**Patient ID**	**Sex**	**Age at onset**	**Seizure classification**	**Etiology**	**Family history**
37	F	7	Generalized tonic-clonic seizures	Unknown	Sister with epilepsy
75	F	47	Focal with impairment of consciousness, involving subjective sensory phenomena, evolving to a bilateral, convulsive seizure	Unknown	Sister with epilepsy
43	F	0.5	Focal with impairment of consciousness, with observable motor or autonomic components, occasionally evolving to a bilateral, convulsive seizure	Unknown	Mother, brother with epilepsy
52	F	62	Focal with impairment of consciousness and with observable motor components	Structural	Brother, sister with DM, sister with HI
53	M	25	Focal with impairment of consciousness, involving subjective sensory phenomena	Unknown	Mother with HI

*DM *diabetes mellitus, *HI *hearing impairment. Seizure classification and assessment of etiology conform to the criteria in the Report of the ILAE Commission on Classification and Terminology [40].

### Nonsynonymous variants with previously reported disease associations

We observed five nonsynonymous mtDNA variants with previously reported disease associations. Three of them were situated in the *MTCO1* gene. The m.6489C > A transversion (p.L196I, *MTCO1*) has been reported in a patient with therapy-resistant epilepsy [41] and in patients with matrilineal diabetes [42]. The second variant m.6253T > C (p.M117T, *MTCO1*) has been reported with an association to prostate cancer [43] and the third variant m.7444G > A (p.*514K, *MTCO1*) in association with Leber’s hereditary optic neuropathy or deafness [44-46]. The remaining two variants were located in *MTND5* including m.12811T > C (p.Y159H) and m.13637A > G (p.Q434R). These variants have been postulated to affect the phenotype of Leber’s hereditary optic neuropathy [47-49]. Prediction algorithms did not support a pathogenic role for these variants.

### Rare mtDNA variants

Four nonsynonymous polymorphisms found in the patients were considered rare (Table 4). Three of them have been previously reported in Finnish individuals. Two of the rare nonsynonymous mutations, m.8923A > G and m.15725C > T, were predicted to be damaging with SIFT BLink and PMut, but not with PolyPhen-2. In addition, a novel m.11392A > G synonymous change in *MTND4* was also discovered in a sample belonging to haplogroup K.

**Table 4:** Rare mtDNA variants discovered in 79 patients with epilepsy

**Variant**	**Gene**	**Amino acid change**	**PolyPhen (%)**^**1**^	**SIFT BLink**^**2**^	**PMut prediction**^**3**^	**PMut reliability score**^**4**^	**Database hits**^**5**^	**Source**
8923A > G	*MTATP6*	p.T133A	1.1	0	Pathogenic	4	3	Finland, Japan
9480T > C	*MTCO3*	p.F92L	38.7	0.51	Pathogenic	6	3	Finland
14564A > G	*MTND6*	p.V37A	0	1	Neutral	0	2	Finland, China^6^
15725C > T	*MTCYB*	p.L327F	0.1	0.03	Pathogenic	4	3	USA, Native American, Kazakhstan
11392A > G^Novel^	*MTND4*	syn	n.a.	n.a.	n.a.	n.a.	0	n.a.

*syn *synonymous variant, *n.a*. not applicable.

^1^http://genetics.bwh.harvard.edu/pph2/ PolyPhen-2 pathogenicity prediction levels: Possibly damaging > 50%, probably damaging > 90%.

^2^http://sift.jcvi.org/www/SIFT_BLink_submit.html SIFT BLink pathogenicity prediction levels: Damaging amino acid substitutions ≤ 0.05.

^3^http://mmb.pcb.ub.es.

^4 ^PMut pathogenicity prediction reliability score, 0 (lowest reliable) to 9 (most reliable).

^5 ^Number of hits among 2704 sequences in mtDB database http://www.mtdb.igp.uu.se/ and among 8813 sequences in HmtDB database http://www.hmtdb.uniba.it accessed in February 2013. Each sequence hit was counted once, if present in both databases.

^6^ A patient with Leber’s hereditary optic neuropathy [49].

GenBank accession: KC763372 - KC763450.

## Discussion

The m.15218A > G (p.T158A, *MTCYB*) variant was found in five patients with epilepsy and with a maternal history of epilepsy, SNHI or diabetes. The patients belonged to haplogroup U5a. Haplogroup U5 has previously been suggested to have possible disease associations, as it has been found to be more frequent in patients with occipital stroke and migrainous stroke [50]. Stroke-like episodes in mitochondrial diseases can resemble occipital epileptic seizures [51] and mitochondrial diseases often manifest themselves only in epileptic seizures [5]. Haplogroup U5a has been associated with a rapid progression to AIDS and death among HIV-1 infected patients, especially those harbouring the m.15218A > G variant [52].

Haplogroup U5 is more common in Finland than the rest of Europe. Its frequency is ~ 30% in the Finnish population compared to 18 - 22% in Western and Eastern Europe [30]. Haplogroup U5 is especially common in Northern Finland and its variation is low there. The reason for this is suspected to be a founder effect of a relatively small settler group that colonized northern Finland after the 16^th^ century [21]. Furthermore, haplogroup U constitutes 32-52% of mtDNA in the indigenous Saami people of Northern Scandinavia and Northwestern Russia. Admixture of Finns and the Saami has been reported in the North of Finland [19,53]. In order to avoid bias caused by geographical differences in haplogroup frequencies we collected our patient and control samples from the same area.

We used three different methods for predicting the pathogenic potential of nonsynonymous mutations. PolyPhen-2 predicted the highest number of benign polymorphisms, whilst SIFT BLink predicted the highest number of pathogenic mutations. PolyPhen-2 has been reported to be sensitive in predicting neutral polymorphisms [54] with a 5% false negative rate for benign variants. On the other hand, SIFT BLink has been reported to be more reliable in detecting truly pathogenic variants. Both PMut and SIFT BLink make use of evolutionary conservation data and yield comparable results, while PolyPhen-2 makes use of physical and chemical properties of amino acids in addition to evolutionary conservation data. We considered the variants that all three algorithms predicted pathogenic to be the best candidates for pathogenic mutations.

The m.15218A > G mutation in *MTCYB* was predicted to be pathogenic by the three algorithms. This mutation leads to substitution of a conserved hydrophilic threonine by a hydrophobic alanine at position 159 of the cytochrome b subunit of complex III. Out of the 60 species compared, 53 species harbour threonine in this position [27] and only Barbary macaque (*Macaca sylvanus*) harbours alanine in this position of the subunit. The cytochrome b subunit of complex III forms the catalytic core of cytochrome bc_1_ complex along with cytochrome c_1_ and Rieske iron-sulphur protein. Changes in this subunit have been shown to cause changes to the catalytic function of complex III, thus leading to complex III defects [55].

The m.15218A > G variant is present in haplogroups M7, M10, HV, H13 and U5a1 [56] indicating that m.15218A > G is a homoplasic variant. We found this variant in two haplotypes within haplogroup U5a1, the major haplotype being identical to that detected previously in Finnish population controls [31] and the minor haplotype being identical to that in a Finnish patient with diabetes mellitus [42]. Evolutionary conservation of the amino acid position, results from the prediction algorithms and the homoplasic nature of the variant suggest that m.15218A > G is mildly deleterious rather than a neutral variant.

The m.14198G > A variant (p.T159M, *MTND6*) found in one patient was also predicted to be damaging. This variant has been found to exist in haplogroups G and H [28,29], although in our patient data it was present in haplogroup U5a1. PMut predicted it to be pathogenic but the reliability score was low and, therefore, we cannot make conclusions of its true pathogenic potential. It is noteworthy though that it was also found in haplogroup U5a1.

Other mutations predicted to be pathogenic by the three algorithms were m.9903T > C (p.F233L, *MTCO3*) and m.9055G > A (p.A177T, *MTATP6*). The m.9033T > C variant occurred in two patients belonging to haplogroup K but was not present in the controls. It is present in one haplogroup L2a sequence [GenBank: EF657335], in two haplogroup K1c1 sequences [GenBank: EU262720, GenBank: EU753433] and in a haplogroup X2 sequence [39] in the HmtDB and mtDB databases [28,29]. We suggest that m.9033T > C is a rare homoplasic variant occurring in haplogroups K, X and L and that its pathogenic nature is uncertain. The five nonsynonymous mutations with previous disease association were not predicted to be deleterious but, interestingly, m.6489C > A (p.L196I, *MTCO1*) has been reported to be associated with therapy-resistant epilepsy [41].

The m.9055G > A (p.A177T, *MTATP6*) mutation is the defining variant for haplogroup K. It was predicted to be damaging by the three algorithms, but was present both in the patients and in the controls. It has been suspected to be deleterious, as an excess of haplogroup K patients has been found to convey a greater risk to lipodystrophy among HIV-patients on antiretroviral therapy [57]. It is interesting that both m.9055G > A and m.9903T > C are associated with haplogroup K. This haplogroup might harbour several mtDNA variants that could impair mitochondrial function.

## Conclusions

In this study, we found that the nonsynonymous mtDNA variant m.15218A > G (p.T158A, *MTCYB*) is more common in epilepsy patients with maternal family members with epilepsy, SNHI or diabetes mellitus. The m.15218A > G variant has previously been suggested to be mildly deleterious, which was supported by our results. We suggest that mildly deleterious mtDNA mutations, such as m.15218A > G, may be considered a risk factor for epilepsy in patients with maternal relatives with SNHI, epilepsy or diabetes mellitus.

## Abbreviations

SNHI: Sensorineural hearing impairment; mtDNA: Mitochondrial DNA; D-loop: Noncoding displacement loop in the mitochondrial DNA; CSGE: Conformation sensitive gel electrophoresis.

## Competing interests

The authors declare that they have no competing interests.

## Authors’ contributions

HKS carried out the laboratory experiments, result analysis and was responsible for writing the manuscript. JSM collected and edited the mtDNA sequence data into sequence files, submitted mtDNA sequences, contributed to the study plan and data analysis. TVK carried out part of the CSGE analysis. SF contributed to the study plan and data analysis. KM planned the study protocol, selected and collected the epilepsy patients and reviewed the manuscript critically. All authors read and approved the final manuscript.

## Pre-publication history

The pre-publication history for this paper can be accessed here:

http://www.biomedcentral.com/1471-2350/14/73/prepub

## Supplementary Material

Additional file 1 **CSGE primer set.** Primer sequences used in PCR fragments for CSGE analysis. Click here for file
